# Effectiveness, Cost-effectiveness, and Cost-Utility of a Digital Smoking Cessation Intervention for Cancer Survivors: Health Economic Evaluation and Outcomes of a Pragmatic Randomized Controlled Trial

**DOI:** 10.2196/27588

**Published:** 2022-03-17

**Authors:** Ajla Mujcic, Matthijs Blankers, Brigitte Boon, Irma M Verdonck-de Leeuw, Filip Smit, Margriet van Laar, Rutger Engels

**Affiliations:** 1 Erasmus School of Social and Behavioural Sciences Erasmus University Rotterdam Netherlands; 2 Trimbos Institute Utrecht Netherlands; 3 Department of Psychiatry Amsterdam University Medical Center, Location AMC University of Amsterdam Amsterdam Netherlands; 4 Department of Research Arkin Mental Health Care Amsterdam Netherlands; 5 Academy het Dorp Arnhem Netherlands; 6 Siza Arnhem Netherlands; 7 Tranzo Tilburg University Tilburg Netherlands; 8 Department of Otolaryngology—Head and Neck Surgery Amsterdam University Medical Center Cancer Center Amsterdam Amsterdam Netherlands; 9 Department of Clinical, Neuro and Developmental Psychology Amsterdam Public Health Research Institute Vrije Universiteit Amsterdam Netherlands; 10 Department of Epidemiology and Biostatistics Amsterdam Public Health Research Institute Amsterdam University Medical Center, Location VUmc Amsterdam Netherlands

**Keywords:** smoking cessation, cancer survivors, effectiveness, cost-effectiveness, eHealth

## Abstract

**Background:**

Smoking cessation (SC) interventions may contribute to better treatment outcomes and the general well-being of cancer survivors.

**Objective:**

This study aims to evaluate the effectiveness, cost-effectiveness, and cost-utility of a digital interactive SC intervention compared with a noninteractive web-based information brochure for cancer survivors.

**Methods:**

A health economic evaluation alongside a pragmatic 2-arm parallel-group randomized controlled trial was conducted with follow-ups at 3, 6, and 12 months. The study was conducted in the Netherlands over the internet from November 2016 to September 2019. The participants were Dutch adult smoking cancer survivors with the intention to quit smoking. In total, 165 participants were included and analyzed: 83 (50.3%) in the MyCourse group and 82 (49.7%) in the control group. In the intervention group, participants had access to a newly developed, digital, minimally guided SC intervention (MyCourse-Quit Smoking). Control group participants received a noninteractive web-based information brochure on SC. Both groups received unrestricted access to usual care. The primary outcome was self-reported 7-day smoking abstinence at the 6-month follow-up. Secondary outcomes were quality-adjusted life years gained, number of cigarettes smoked, nicotine dependence, and treatment satisfaction. For the health economic evaluation, intervention costs, health care costs, and costs stemming from productivity losses were assessed over a 12-month horizon.

**Results:**

At the 6-month follow-up, the quit rates were 28% (23/83) and 26% (21/82) in the MyCourse and control groups, respectively (odds ratio 0.47, 95% CI 0.03-7.86; *P=*.60). In both groups, nicotine dependence scores were reduced at 12 months, and the number of smoked cigarettes was reduced by approximately half. The number of cigarettes decreased more over time, and the MyCourse group demonstrated a significantly greater reduction at the 12-month follow-up (incidence rate ratio 0.87; 95% CI 0.76-1.00; *P=*.04). Intervention costs were estimated at US $193 per participant for the MyCourse group and US $74 for the control group. The mean per-participant societal costs were US $25,329 (SD US $29,137) and US $21,836 (SD US $25,792), respectively. In the cost-utility analysis, MyCourse was not preferred over the control group from a societal perspective. With smoking behavior as the outcome, the MyCourse group led to marginally better results per reduced pack-year against higher societal costs, with a mean incremental cost-effectiveness ratio of US $52,067 (95% CI US $32,515-US $81,346).

**Conclusions:**

At 6 months, there was no evidence of a differential effect on cessation rates; in both groups, approximately a quarter of the cancer survivors quit smoking and their number of cigarettes smoked was reduced by half. At 12 months, the MyCourse intervention led to a greater reduction in the number of smoked cigarettes, albeit at higher costs than for the control group. No evidence was found for a differential effect on quality-adjusted life years.

**Trial Registration:**

The Netherlands Trial Register NTR6011; https://www.trialregister.nl/trial/5434

**International Registered Report Identifier (IRRID):**

RR2-10.1186/s12885-018-4206-z

## Introduction

### Background

In cancer survivors, continued tobacco use is one of the most important risk factors for the development of secondary cancers, iatrogenic effects of cancer treatment, and cancer mortality [1]. The prevalence of smoking among cancer survivors is considerable, estimated at 11.8% for US cancer survivors in 2018 [2], with rates that tend to be higher among women and younger cancer survivors [3,4] and those with low health-related quality of life [5]. In the Netherlands, no difference in smoking prevalence was found between cancer survivors and noncancer survivors after adjusting for sociodemographic variables [6].

Many cancer centers in the United States have not implemented tobacco treatment services [7]; less than half of cancer care providers routinely discuss smoking cessation (SC) medication with cancer survivors [8]; and the delivery of effective SC support to cancer survivors is currently lacking [9,10]. In Europe, the general picture is comparable [11]. At the same time, cancer survivors are generally receptive toward discussions of SC with their health care professionals [3,12,13]. Among patients with head and neck cancer receiving SC counseling, 26% higher SC rates were observed than control groups in a meta-analysis of 3 randomized controlled trials (RCTs) and 3 cohort studies [14]. Distance-based SC support was also found to be more effective in reducing smoking than a range of control conditions [15]. Nayan et al [16] reported that SC interventions delivered in the perioperative period lead to higher quit rates in cancer survivors (odds ratio [OR] 2.31) but found no effect of SC interventions delivered in the cancer clinic. In addition, when considering biochemically validated smoking status, no significant effect of SC interventions was found in cancer survivors [17]. An integrated tobacco treatment program in a cancer setting showed that high abstinence rates of 45.8% at 6 months could be achieved, as demonstrated in a cohort study of 3245 patients (593 had no cancer history) [18], but this was a highly intensive treatment program consisting of in person and telephone sessions spanning 8 to 12 weeks, which not only provided behavioral counseling for SC but also pharmacotherapy and treatment of related mental health conditions. Overall, there is a paucity of literature on SC interventions, specifically for cancer survivors, and the relevant literature shows conflicting outcomes.

Even fewer studies have evaluated the cost-effectiveness of digital SC interventions in the population of cancer survivors. Digital interventions may have the benefit of being scalable, easily accessible, and providing a cost-effective way to support the growing number of cancer survivors [19]. A pilot study demonstrated good acceptability of a digital SC intervention among cancer survivors [20]. A recent meta-analysis [15] indicated that few SC interventions for cancer survivors were digital interventions (2 out of 10), with most being delivered over the telephone. However, the effectiveness and cost-effectiveness of existing digital SC interventions over the internet when specifically tailored to cancer survivors is unclear.

### Objectives

**Figure 1:**
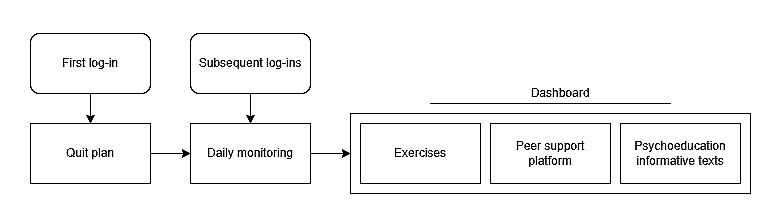
Intervention flowchart (adapted from Mujcic et al [21]).

It was deemed timely and appropriate to launch a new study evaluating the effectiveness and cost-effectiveness of a recently developed digital intervention with minimal guidance aimed at supporting cancer survivors to quit smoking: MyCourse—Quit Smoking (in Dutch: MijnKoers—Stoppen met Roken; Figure 1 [21]). Details of how the intervention was developed are provided elsewhere [21]. In this study, we aim to answer the following research questions:

1. Is the digital interactive SC intervention *MyCourse—Quit Smoking* more effective than a web-based SC brochure to improve smoking cessation rates?
2. Is the digital interactive SC intervention *MyCourse—Quit Smoking* more cost-effective than a web-based SC brochure in terms of incremental costs per reduced pack-year and incremental costs per quality-adjusted life year (QALY) gained?

## Methods

### Design

The effectiveness, cost-effectiveness, and cost-utility of a digital SC intervention for cancer survivors was evaluated in an individual RCT with 2 parallel arms. The trial was conducted in the Netherlands between 2016 and 2019. The first inclusion was on November 4, 2016, and the last inclusion was on September 15, 2018. The last follow-up data were collected on September 24, 2019. The study was prospectively registered in the Netherlands Trial Register (NTR6011) on September 1, 2016. For an extensive description of the study protocol, see the study by Mujcic et al [21]. This study was part of a set of 2 separate RCTs on interventions for SC and alcohol moderation, both targeting cancer survivors. The results of the RCT on the alcohol moderation intervention (MyCourse—Moderate Drinking) will be published separately.

### Ethics Approval

Ethical approval was obtained from an accredited medical research and ethics committee in the Netherlands (Toetsingscommissie Wetenschappelijk Onderzoek Rotterdam e.o. NL55921.101.16).

### Participants and Recruitment

**Figure 2:**
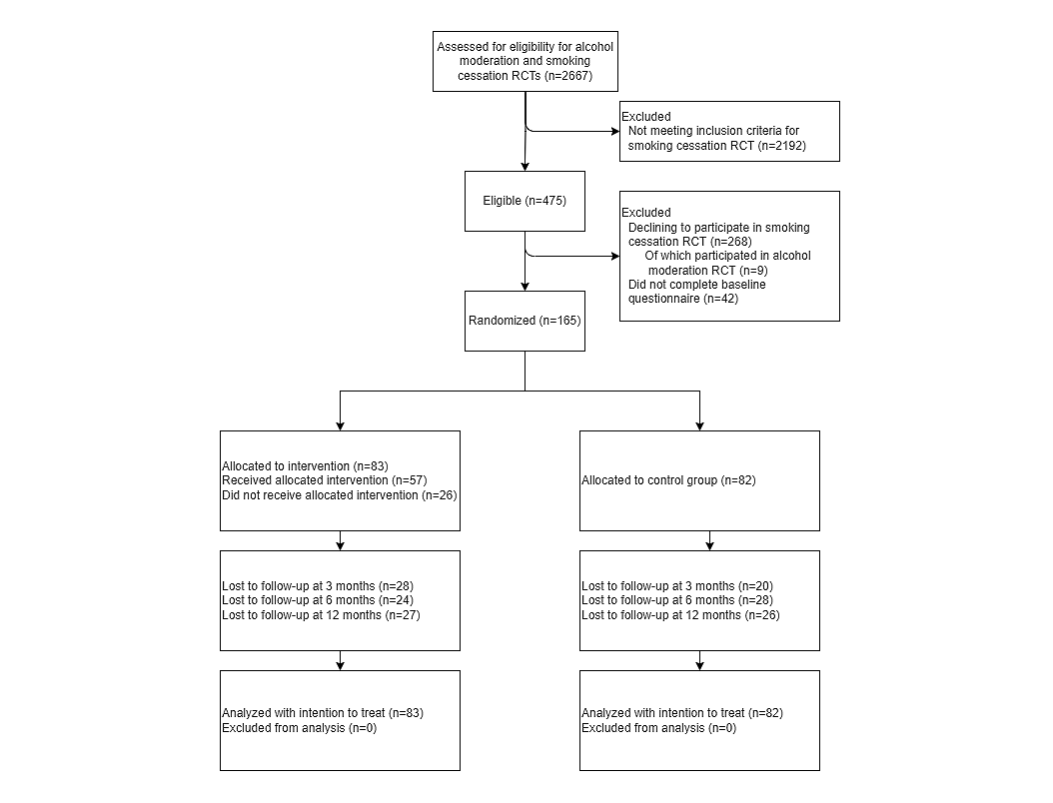
CONSORT (Consolidated Standards of Reporting Trials) flowchart. RCT: randomized controlled trial.

A dedicated website was created where participants could inform themselves about the study and apply for participation. Applicants for the trial were eligible if they were aged ≥18 years, diagnosed with any form of cancer in the past 10 years, had a PC or laptop and internet connection at home, had the ability and intention to participate in the 12-month study, smoked ≥5 cigarettes per day in the past 7 days, and had the intention to quit smoking cigarettes. Those who had insufficient mastery of the Dutch language; those who were pregnant; or those who self-reported suicidal ideation, acute psychosis, severe alcohol dependence, dementia, or severe depression were excluded. These criteria were assessed using a web-based screening questionnaire on the website. The same screening questionnaire was used for both trials to evaluate the SC and alcohol moderation intervention [21]. Some people were eligible for both the current SC trial and the alcohol moderation trial; they were offered to participate in 1 trial of their choice (Figure 2). None of the participants were allowed to participate in both trials simultaneously.

Recruitment was conducted through web-based and offline strategies. Targeted web-based (social) media and search engine advertisements were pointed to the website and web-based screening questionnaire. SC clinics, oncology departments, and meeting centers for cancer survivors were contacted and offered promotional material (flyers and posters) to help refer cancer survivors to the website.

### Procedure

After completing the screening questionnaire on the study’s website, applicants were informed by a computer-generated email about their eligibility for study participation. Those eligible were sent an invitation email containing patient information, an informed consent form, and a link to register. They then had 30 days to decide on their participation; during this time, they could contact the research team or an independent physician with questions. Once the informed consent form was digitally signed, the participants completed the baseline questionnaire. Immediately after completion of the baseline measurement, participants were allocated to either the intervention or the control group arm in a 1:1 ratio through adaptive randomization (minimization of baseline imbalance for age, sex, and education level) performed automatically by a server-side PHP script using a Mersenne twister random number generator. Participants received an email confirming their allocation and containing their username and instructions on how to log on. The participants were not blinded to the study group allocation. At 3, 6, and 12 months after randomization, participants received a link to the web-based questionnaire via email. Nonresponders received up to 3 reminder emails and, in case of continued nonresponse, were contacted by telephone. For each completed follow-up assessment, they were reimbursed approximately US $30. As this was a pragmatic randomized controlled trial, in both groups, patients were allowed to use additional support (eg, nicotine replacement therapy) if they felt they needed it.

### Intervention

MyCourse—Quit Smoking is a newly developed, minimally guided, digital intervention aimed at supporting SC in cancer survivors, based on well-established therapeutic approaches: motivational interviewing, cognitive behavioral therapy, and acceptance and commitment therapy. These approaches have been incorporated into effective SC interventions in the general population [22-24]. Cancer survivors and professionals in eHealth, oncology, and SC were involved throughout the development process. The intervention was accessible through a PC, tablet, and smartphone. At first, log-in participants were guided in setting up a quit plan including a quit date, after which they gained access to 13 exercises, a web-based diary for self-monitoring of tobacco use and contextual cues, information on SC and cancer, and a peer support platform (Figure 1 [21] and Multimedia Appendix 1). Participants could choose to use the intervention whenever they wanted for the duration of the study but were encouraged to log in daily for at least 4 weeks. Elsewhere, we have provided a more extensive description of the intervention and its development [21].

### Control Group

Participants in the control group received access to a noninteractive web-based information brochure on the risks of smoking and tips on how to quit smoking, which they could access whenever they wanted by logging into the website. It contained both general SC information and information specifically relevant to cancer survivors. However, it did not contain any responsive elements of the MyCourse intervention.

### Additional Support

Participants in both groups were free to seek additional help if needed and were referred to the National SC Information Line (in Dutch: *Rokeninfo-lijn*) for more information. The use of additional support was retrospectively assessed at follow-up. At the end of the study, at 12 months after randomization, the control group participants also received access to the digital intervention MyCourse—Quit Smoking.

### Measures

#### Baseline

Sociodemographic characteristics and type of cancer were assessed. Tobacco use was assessed by using Timeline Followback (TLFB) self-reports [25] for the number of cigarettes smoked in the past 7 days. Nicotine dependence was assessed using the Fagerström Test for Nicotine Dependence (FTND) [26], a 6-item questionnaire. In participants reporting alcohol use, problematic alcohol use was assessed using the Alcohol Use Disorders Identification Test (AUDIT) [27], a 10-item questionnaire. The Marlowe-Crowne Social Desirability Scale (MCSDS) was used to assess the reliability of the self-reported questionnaire data [28]. QALYs were assessed using the 5-level EuroQol (EQ-5D-5L) [29]. In addition, the Medical Outcomes Study Short Form Survey-36 was administered to calculate the Short Form 6-dimension (SF-6D) quality of life measure [30] using the Brazier algorithm [31].

#### Follow-up Measurements

At all follow-up measurements, we assessed tobacco and alcohol use by using TLFB self-reports, nicotine dependence by using FTND, productivity and health care costs, QALYs using EQ-5D-5L and Medical Outcomes Study Short Form Survey-36, and the use of other SC support and e-cigarettes. Intervention use variables (eg, number of log-ins and use of major content elements) were collected throughout the study period. At 3-month follow-up treatment, satisfaction was assessed using the German adapted Client Satisfaction Questionnaire (Fragebogen zur Messung der Patientenzufriedenheit, ZUF-8), which was translated into Dutch [32].

#### Primary and Secondary Outcome Measures

The primary predefined endpoint was 7-day smoking abstinence at the 6-month follow-up, measured by using TLFB self-reports. Those who reported not smoking at all in the past 7 days were considered abstinent smokers (yes or o). Secondary measures included the number of smoked cigarettes in the past 7 days, nicotine dependence as measured by the FTND (range 0-10), treatment satisfaction as measured by ZUF-8 (range 8-32), health care costs, productivity loss, and QALYs.

#### Costs

Costs were calculated from a societal perspective for the year 2019. Intervention costs included depreciation costs (the estimated loss of value of an interactive website, as it needs to be updated regularly to keep up with technological advancements and prevent safety issues), costs for hosting the website, and technical support and recruitment costs (which consisted of both advertising costs in web-based and offline media, as well as the costs of printing promotional material), and these were allocated evenly to all participants regardless of intervention use. Recruitment costs were included as they were considered an essential part of the intervention and control condition. Health care costs were calculated by multiplying all reported contacts with health services with the standard unit cost prices for the Netherlands [33]. Health service costs stemmed from contacts with specialized somatic and mental health care and patients’ out-of-pocket costs for home care, but travel costs were not included because in both groups the interventions were delivered over the internet. Other health care costs included appointments for physiotherapy, alternative medicine, and social work. Medication costs were valued by multiplying the reported dose of that drug with unit cost price [34]. Productivity loss included costs from absenteeism and presenteeism, calculated according to the friction cost method, meaning productivity losses were limited to a maximum of 85 days after which production losses cease to exist because the sick employee would be replaced by another and using an elasticity factor of 0.8 because there is not a strict 1:1 relation between days not worked and productivity losses. Cost data related to health care use and productivity loss were assessed using the Trimbos and iMTA questionnaire for Costs associated with Psychiatric Illness [35]. Cumulative societal costs over the entire follow-up period of 12 months were calculated as the sum of health care costs and productivity losses. Costs were converted from euros to US dollars using purchasing power parities for the reference year 2019.

### Sample Size

A study on an SC intervention among cancer survivors found a quit rate of 30% in the active SC intervention group versus 15% in the SC control group, translating to a relative risk (RR) of 2.14 [36]. A pilot trial of an acceptance and commitment theory web-based SC intervention found a 23% quit rate in the experimental arm versus a 10% quit rate in the control arm, translating to an RR of 2.20 [24]. On the basis of the average of these RRs, an RR of 2.1 was expected, translating into a 21% quit rate in the experimental arm, assuming a 10% quit rate in the control arm at the 6-month follow-up. On the basis of the conventional statistical significance level (Cronbach *α*≤.05), an RR of 2.1 at the 6-month follow-up, 204 participants would yield a power of 0.83 for 1-sided tests or a power of 0.74 for 2-sided tests.

### Statistical Analyses

#### Imputation of Missing Data

All primary and secondary outcome measures were analyzed in accordance with the intention-to-treat principle, except for ZUF-8 (treatment satisfaction). To that end, missing data for primary and secondary outcome measures and costs were imputed using the predictive mean matching method from the mice package in R (R Foundation) [37]. The responses to ZUF-8 were not imputed. For the 2 deceased participants, the smoking status and number of cigarettes smoked were left missing, and the quality of life (EQ-5D-5L) score and costs were set to 0.

#### Effect Evaluation

Tobacco abstinence (binary yes or no outcome) was analyzed using a Generalized Linear Mixed Model (GLMM) with a binomial distribution and log link function. Although imputation of missing values is not always deemed necessary when running a GLMM, imputation of missing values before running a GLMM allowed us to consider all variables that could have impacted dropout and not only the variables within a specific model. The number of cigarettes smoked (count data 0, 1, …, N) was analyzed using a GLMM with a log link function and negative binomial distribution [38]. Included covariates were the minimized variables (gender, age, and education) and the MCSDS (social desirability of responses). Model estimates, ORs, incidence rate ratios (IRRs) or Cohen *d*, 95% CIs, and *P* values were reported. The effect of time on the number of cigarettes was analyzed using an *F* test. Differences between the intervention and control groups on FTND nicotine dependence and ZUF-8 patient satisfaction scores were analyzed using a Linear Mixed Model for the Gaussian distribution with identity as the link function; estimates, 95% CIs, and *P* values were also reported.

#### Cost-effectiveness Analyses

An economic evaluation was conducted alongside this RCT following the approach of Drummond et al [39] and in concordance with the Consolidated Health Economic Evaluation Reporting Standards statement [40]. QALYs over the entire follow-up period were computed using the Dutch tariff (utility weights) [41] through the area under the curve method; that is, linear interpolation for cumulating the cost over the 12-month follow-up period. The incremental cost-effectiveness ratio (ICER) was calculated as follows: ICER=(C_1_−C_0_)/(E_1_−E_0_), where C refers to costs, E refers to effect, and the subscripts 1 and 0 refer to the MyCourse and control groups, respectively. We generated 2500 replicate samples by bootstrap and estimated the corresponding incremental costs and effects for each replicate sample, which were then plotted on a cost-effectiveness plane. In addition to the ICER per QALY gained, the ICER per reduced pack-year was calculated. Pack-years were calculated by multiplying cigarettes smoked in the past week by 52 (weeks in a year) and dividing by 20 (cigarettes per pack) and 365 (days in a year). We calculated ICERs from the following four perspectives: societal, health care, productivity loss, and intervention cost–only. Cost-effectiveness acceptability curves (CEACs) were graphed to assess the likelihood that the intervention was deemed cost-effective, given a series of willingness-to-pay ceilings for gaining 1 QALY.

#### Sensitivity Analyses

The negative binomial and binomial analyses of the mice-imputed data constituted the main analyses. We conducted several sensitivity analyses for the effectiveness and incremental cost-effectiveness analyses using QALYs based on the SF-6D (instead of the EQ-5D-5L), using Winsorizing cost outliers, using the Amelia-2 package (R Foundation) instead of the mice-package for imputations, considering a gradual decline in pack-years, and using different statistical models (Multimedia Appendix 1).

## Results

### Sample Characteristics

The participant flow and retention rates are shown in Figure 2. Of the 2192 ineligible people, 1684 (76.82%) had no diagnosis of cancer in the past 10 years. Of the 475 eligible cancer survivors, 268 (56.4%) declined to participate, 9 (3.4%) of whom chose to participate in our parallel RCT on MyCourse for alcohol moderation, and another 42 (8.8%) cancer survivors did not complete the baseline questionnaire and were, therefore, not randomized. Sociodemographic and other characteristics are reported in Table 1. The participants’ mean age was 54.2 (SD 11.2) years, 17.6% (29/165) were men, approximately half were married or living together (93/165, 56.4%), and 30.3% (50/165) had a lower education level. On average, participants had smoked for 34.5 (SD 12.0) years and smoked 100 (SD 51.2) cigarettes per week. Three participants quit smoking between screening and completing the baseline questionnaire (Table 2). Breast cancer (75/165, 45.5%), lung cancer (23/165, 13.9%), uterus cancer (19/165, 11.5%), and head and neck cancer (18/165, 10.9%) were the most frequently reported. There was no difference in the proportion of missing data between groups at any of the time points (*χ*^2^_1_=0.09; *P=*.77; Multimedia Appendix 2).

**Table 1:** Baseline characteristics of study participants.^a^

		MyCourse (n=83)	Control (n=82)	Total (N=165)
**Gender, n (%)**				
	Women	70 (84)	66 (80)	136 (82.4)
	Men	13 (16)	16 (20)	29 (17.6)
Age (years), mean (SD)		55.0 (12.1)	53.3 (10.3)	54.2 (11.2)
**Education, n (%)**				
	Higher level	25 (30)	19 (23)	44 (26.7)
	Midlevel	33 (40)	38 (46)	71 (43.0)
	Lower level	25 (30)	25 (30)	50 (30.3)
**Marital status, n (%)**				
	Married or living together	47 (57)	46 (56)	93 (56.4)
	Unmarried or living alone	15 (18)	11 (13)	26 (15.8)
	Divorced	16 (19)	20 (24)	36 (21.8)
	Widowed	5 (6)	5 (6)	10 (6.1)
**Smoking behavior, mean (SD)**				
	Years smoked	34.4 (11.8)	34.6 (12.2)	34.5 (12.0)
	Number of cigarettes in the past 7 days	101.8 (54.3)	98.2 (48.2)	100 (51.2)
	FTND^b^	4.9 (2.4)	4.9 (2.3)	4.9 (2.4)
**Drinking behavior**				
	Drank alcohol in the last month, n (%)	55 (66)	55 (67)	110 (66.7)
	Number of drinks in the past 7 days, mean (SD)	6.9 (13.1)	5.6 (8.7)	6.2 (11.2)
	AUDIT,^c^ mean (SD)	3.7 (5.1)	3.6 (4.2)	3.6 (4.7)
**Cancer diagnosis, n (%)**				
	Breast	42 (51)	33 (40)	75 (45.4)
	Lung	14 (17)	9 (11)	23 (13.9)
	Uterus	7 (8)	12 (15)	19 (11.5)
	Head and neck	10 (12)	8 (10)	18 (10.9)
	Colon	5 (6)	5 (6)	10 (6.0)
	Other (including bladder, lymphatic, melanoma, skin, kidney, prostate, etc)	5 (6)	26 (32)	20 (12.1)

^a^Percentages may not add up to 100 because of rounding.

^b^FTND: Fagerström Test for Nicotine Dependence.

^c^AUDIT: Alcohol Use Disorders Identification Test.

**Table 2:** Smoking behavior outcomes and treatment effects (missing data were imputed; a total of 3 participants quitted smoking between screening and completing the baseline questionnaire).

Variable		MyCourse (n=83)	Control (n=82)	Effect size (95% CI)
**Cessation, n (%)^a^**				
	Baseline	2 (2.4)	1 (1.2)	N/A^b^
	3 months	18 (21.7)	19 (23.2)	Adjusted OR^c^ 0.33 (0.02 to 5.44)
	6 months	23 (27.7)	21 (25.6)	Adjusted OR 0.47 (0.03 to 7.86)
	12 months	27 (32.6)	23 (28.1)	Adjusted OR 0.58 (0.03 to 9.78)
**Number of cigarettes, mean (SD)^d^**				
	Baseline	101.8 (54.3)	98.2 (48.2)	N/A^b^
	3 months	54.3 (51.1)	54.2 (48.2)	Adjusted IRR^e^ 0.95 (0.85 to 1.06)
	6 months	50.5 (50.5)	50.1 (47.5)	Adjusted IRR 0.96 (0.85 to 1.08)
	12 months	45.4 (50.9)	49.6 (44.9)	Adjusted IRR 0.87 (0.76 to 1.00)^f^
**FTND,^g^ mean (SD)^h^**				
	Baseline	4.9 (2.4)	4.9 (2.3)	N/A^b^
	3 months	2.9 (2.5)	2.8 (2.5)	Cohen *d*=0.03 (−0.27 to 0.34)
	6 months	2.6 (2.6)	2.8 (2.6)	Cohen *d*=0.07 (−0.23 to 0.38)
	12 months	2.4 (2.6)	2.7 (2.5)	Cohen *d*=0.13 (−0.18 to 0.43)

^a^Adjusted coefficients are based on a binomial mixed model with random intercept in which the outcome measure at follow-up is regressed on the baseline number of cigarettes, covariates, and condition.

^b^N/A: not applicable.

^c^OR: odds ratio.

^d^Adjusted coefficients are based on a negative binomial mixed model with random intercept in which the outcome measure at follow-up is regressed on the baseline number of cigarettes, covariates, and condition.

^e^IRR: incidence rate ratio.

^f^Adjusted coefficients are based on a linear mixed model with random intercept in which the outcome measure at follow-up is regressed on the baseline number of cigarettes, covariates, and condition.

^g^FTND: Fagerström Test for Nicotine Dependence.

^h^*P*<.05 (*P*=.04).

### Treatment Uptake and Satisfaction

Overall satisfaction with the SC intervention was highest in the MyCourse group (mean 21.4, SD 4.6) than in the control group (mean 17.3, SD 6.1; Cohen *d*=0.77; *t*_108.8_=4.13; *P*<.001; Multimedia Appendix 2). Most participants in the MyCourse group logged in at least once (57/83, 69%). The number of times participants logged in was skewed, with an average of 20 (SD 61.2) and a median of 3 (range 0-384). The average time between the first and last log-in for those who logged in at least once was 105.2 (SD 157.5; median 24) days. Most reported SC support in addition to MyCourse at the 6-month follow-up was nicotine replacement therapy (control group: 25/82, 30%; MyCourse group: 14/83, 17%) and contact with a health care professional (control group: 7/82, 9%; MyCourse group: 3/83, 4%). The use of nicotine replacement therapy was reported more often (18.1% vs 30.5% at 12 months) in the control group than in the MyCourse group (*P*=.02).

### Adverse Events

Two deaths occurred in the MyCourse group over the course of the study period, which was reported to the medical research and ethics committee. The cause of death was deemed to be unrelated to the study. No other adverse events were observed.

### Incremental Effects

#### Primary Outcome

At the 6-month follow-up, 28% (23/83) of smokers had quit smoking in the MyCourse group versus 26% (21/82) in the control group (Table 2). No difference in 7-day abstinence was found between the 2 groups (adjusted OR 0.47, 95% CI 0.03-7.86; *P=*.60) when controlling for social desirability, baseline number of cigarettes used in the last week, gender, age, and education.

#### Secondary Outcomes

**Figure 3:**
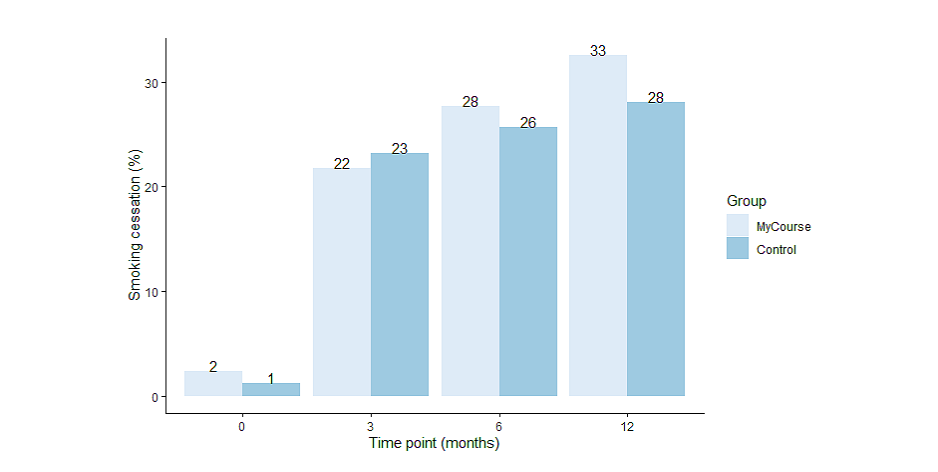
Percentage of quitters in both groups at baseline and during the course of the study. A total of 3 participants quitted smoking between screening and completing the baseline questionnaire.

**Figure 4:**
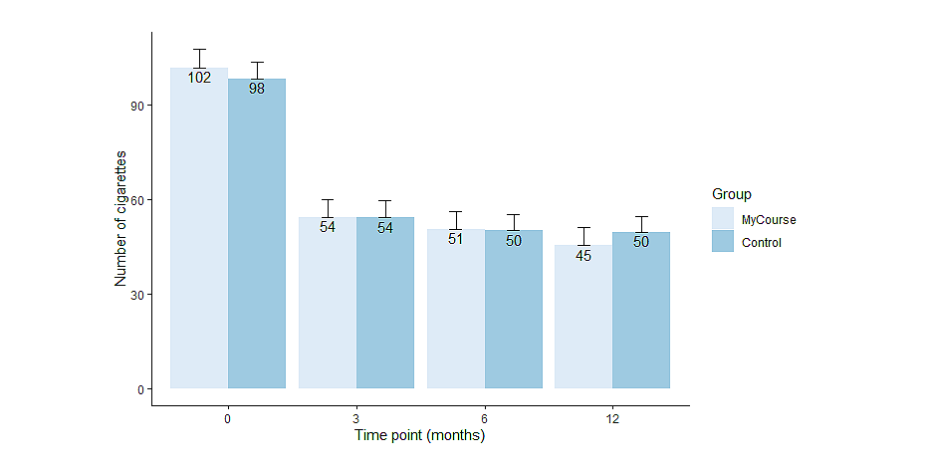
Mean number of cigarettes smoked in both groups at baseline and during the course of the study, including SEs. Error bars show SEs of the mean.

Table 2 and Figure 3 present the effect estimates on the secondary outcomes. In brief, the number of cigarettes smoked in the past week was significantly reduced at all follow-ups and in both groups compared with baseline (*F_3_*=51.5; *P*<.001; Table 2 and Figure 3). At 12-month follow-up, number of cigarettes was reduced by about half in both the MyCourse group, showing an average reduction of 57 cigarettes (57/101.8, 56%), and in the control group, showing an average reduction of 48 cigarettes (48/98.2, 49%; Table 2 and Figure 4). At 12-month follow-up, the reduction of number of cigarettes smoked was significantly greater in the MyCourse group than in the control group (adjusted IRR 0.87, 95% CI 0.76-1.00; *P=*.04). At 3- and 6-month follow-up, the difference in number of cigarettes between groups was not significant.

FTND scores were significantly lower at all follow-ups in both groups compared with baseline scores, whereas the time × condition interaction was not significant (Cohen *d*=0.03; 95% CI −0.27 to 0.34; *P=*.95), indicating no significant difference between the groups over time.

The mean EQ-5D-5L QALYs gained in the intervention group was 0.75 (SD 0.18) and in the control group was 0.78 (SD 0.15). There was no significant effect of treatment on quality of life based on EQ-5D-5L scores (B=−0.03, SE 0.03; *P*=.26).

### Incremental Costs

Table 3 presents the costs per group and the incremental costs (cost difference between the MyCourse and control groups) per cost item. The intervention costs were estimated at US $193 per participant in the MyCourse group and US $74 per participant in the control group. The average health care costs accumulated over the full 12 months follow-up time were US $14,416 (SD US $20,604) per participant in the MyCourse group and US $12,950 (SD US $17,704) per participant in the control group, resulting in incremental health care costs of US $1466 (SD US $27,165). Cost owing to productivity losses was mainly driven by absenteeism at US $10,444 (SD US $17,277) in the MyCourse group and US $8145 (SD US $15,750) in the control group, with high within-group variance. Incremental productivity costs per participant were on average US $1908 (SD US $23,490). The average cumulative societal costs were US $3493 (SD US $38,913) higher in the MyCourse group than in the control group. See Table 3 for a detailed breakdown of the main cost items and the corresponding SDs.

**Table 3:** Mean cumulative costs (in US $) by group and incremental costs.

Cost item			MyCourse; (n=83), mean (SD)		Control; (n=82), mean (SD)	Incremental costs^a^; mean (SD)
**Health care costs**			14,416 (20,604)		12,950 (17,704)	1466 (27,165)
	Specialized somatic	8418 (11,792)		7180 (10,674)		1238 (15,906)
	Specialized psychiatric	2151 (10,143)		1380 (5441)		771 (11,510)
	Patient and family costs	1310 (10,034)		1954 (9252)		−644 (13,648)
	Other	1533 (2671)		1411 (2518)		122 (3671)
	Medication	1358 (3901)		1023 (3254)		335 (5080)
**Productivity loss**			10,720 (17,345)		8812 (15,841)	1908 (23,490)
	Presenteeism	231 (733)		332 (864)		−101 (1133)
	Absenteeism	10,444 (17,277)		8145 (15,750)		2299 (23,379)
	Unpaid work	451 (1048)		474 (1007)		−23 (1453)
Intervention costs			193 (0)		74 (0)	119 (0)
Total societal costs			25,329 (29,137)		21,836 (25,792)	3493 (38,913)

^a^Costs in the MyCourse group minus costs in the control group.

### Cost-Utility

**Figure 5:**
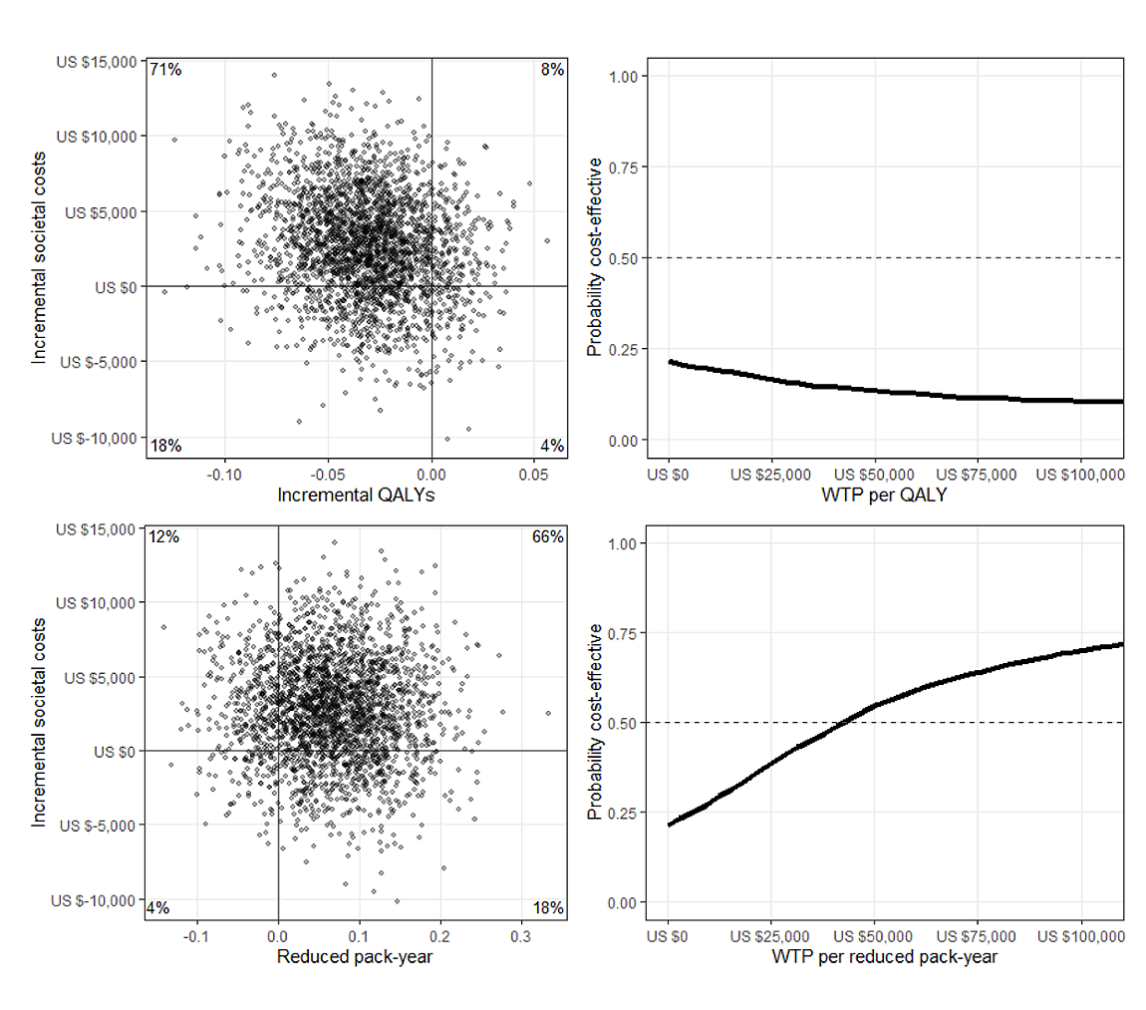
Cost-effectiveness planes and cost-effectiveness acceptability curves in US $. Each quadrant in the cost-effectiveness planes represents a different association between the incremental costs (y-axis) and the incremental effects (x-axis) of the MyCourse group compared with the control group. When incremental cost-effectiveness ratios (ICERs) fall in the upper-right quadrant, this represents more effect at higher costs. When ICERs fall in the upper-left quadrant, this represents less effect at higher costs, meaning the MyCourse group is dominated by the control group. ICERs in the lower-right quadrant represent more effect at lower costs: the dominant quadrant. ICERs in the lower-left quadrant represent less effect at lower costs. QALY: quality-adjusted life year; WTP: willingness to pay.

Participants in the MyCourse group gained fewer QALYs than participants in the control group (0.75 vs 0.78). In addition, the societal costs in the MyCourse group were higher than those in the control group (US $25,329 vs US $21,836). In other words, fewer QALYs were gained against higher costs, which rendered the control group the preferred option, as seen from a cost-effectiveness viewpoint. The cost-effectiveness plane in Figure 5 shows that there is a 71% likelihood that MyCourse is dominated by the control group.

### Cost-effectiveness

Participants in the MyCourse group reduced pack-years more than the control group (0.41 vs 0.34) against higher societal costs (US $25,329 vs US $21,836). Comparing the difference in total societal costs to the difference in pack-years yielded an ICER of US $52,067 per reduced pack-year (95% CI US $32,515-US $81,346). There is a 66% chance that the intervention leads to more reduced pack-years at a higher cost (Figure 5). The CEAC based on the societal cost perspective presented in Figure 5 indicates that the intervention will be preferred over the control group when the willingness to pay per reduced pack-years is more than US $50,000. From an intervention cost–only perspective, ICER per pack-year was calculated to be US $1772 (95% CI US $1384-US $2502). See Table 4 for a breakdown by perspective.

**Table 4:** Incremental cost-effectiveness ratios between baseline and the 12-month follow-up.^a^

Perspective	Incremental costs per reduced pack-year	
	Mean (US $)	95% CI
Health care perspective	21,851	9179-38,920
Productivity loss perspective	28,444	16,832-44,749
Intervention cost–only perspective	1772	1384-2502
Societal perspective	52,067	32,515-81,346

^a^The incremental cost-effectiveness ratios were calculated as follows: (C_1_ – C_0_) / (E_1_ – E_0_), where C refers to costs, E refers to effects, and the subscripts 0 and 1 refer to the experimental and control arms, respectively.

### Sensitivity Analyses

Sensitivity analysis of the Amelia 2-imputed data (adjusted OR 3.16, 95% CI 0.17-57.80; *P=*.44) and completers only (ie, those who completed the questionnaires, without imputation; adjusted OR 2.89, 95% CI 0.16-53.18; *P=*.47) showed similar results on the effect of treatment group on tobacco abstinence rates. The Poisson model with correction for overdispersion showed similar results on the effect of treatment on cigarettes smoked at the 12-month follow-up (adjusted B=−0.c55, 95% CI −1.05 to −0.04; *P=*.03). Sensitivity analyses of the Amelia-2 imputed data also showed a large effect of time but found no effect of treatment group at the 12-month follow-up (adjusted IRR 0.997, 95% CI 0.82-1.22; *P=*.97). The completers only sensitivity analysis showed a greater reduction in the number of cigarettes in the control group at 6 months (adjusted IRR 1.08, 95% CI 1.01-1.15; *P=*.03), but at 12 months, a greater reduction in the MyCourse group (adjusted IRR 0.88, 95% CI 0.82-0.94; *P*<.001). For the number of cigarettes smoked, the social desirability score was a significant predictor in the Poisson model, but not in the negative binomial model. When QALYs were based on SF-6D scores, results of the economic evaluation remained similar. When Winsorization of extreme costs was applied at the 95th percentile, the cost-effectiveness planes and CEACs remained similar, but ICER per pack-year was lower at US $31,342 (95% CI US $17,912-US $50,007). When the gradual decline in the number of pack-years was accounted for, ICER per pack-year was higher (US $68,267, 95% CI US $42,293-US $111,044) and the cost-effectiveness planes comparable (Multimedia Appendix 3). Overall, the sensitivity analyses attested to the robustness of the findings in the main analysis.

## Discussion

### Principal Findings

This study evaluated the effectiveness and cost-effectiveness of MyCourse, a digital SC intervention tailored to cancer survivors, versus a web-based noninteractive information brochure. In the MyCourse group, 27.7% of the participants quit smoking after 6 months. In the control group, 25.6% of the participants quit smoking. The number of cigarettes smoked in the past 7 days was reduced by more than half in both groups. At the 12-month follow-up, MyCourse participants showed significantly larger reductions in the number of smoked cigarettes than participants in the control group. However, no statistically significant difference was found in the SC rates between the intervention and control groups. Nicotine dependence as measured by FTND was also significantly reduced at all time points in both groups, but no difference was found between the groups. Participants in the MyCourse group had significantly higher treatment satisfaction scores than those in the control group. From a societal perspective, the MyCourse intervention was dominated by the control group in the cost-utility analysis. In the cost-effectiveness analysis, MyCourse led to marginally better results against higher costs, with a mean ICER of US $52,067 per reduced pack-year. Cessation rates were high in the MyCourse and control groups.

### Findings in Context

We found no difference in SC rates between the MyCourse and control groups. In previous literature, digital SC interventions have shown superior effectiveness over nonactive control groups (including both usual care and printed self-help materials), among the general population as well as other target groups [42,43]. There is evidence that among cancer survivors, distance-based SC interventions (including digital interventions) also show greater effectiveness than control groups [15]. At the same time, cessation rates in the MyCourse group found in this study are comparable with cessation rates found in 2 previous studies on digital SC interventions for (childhood) cancer survivors [20,44], but cessation rates in our control group were higher than in these previous studies.

Our study did not find an effect on QALYs; a longer follow-up period may be necessary to detect improvements in quality of life among cancer survivors, as their quality of life may be more directly influenced by factors pertaining to cancer diagnosis [45]. There are also some differences in the ICERs that we found compared with previous studies. A systematic review in the Netherlands showed greater cost-utility (< US $22,689 [€20,000] per QALY per year) of intensive SC counseling and pharmacotherapy over care as usual in another patient population: patients with chronic obstructive pulmonary disease [46], and a similar study on a digital SC intervention among the general population showed an ICER of about US $3398 (€3000) per abstinent smoker [47]. The large ICER per reduced pack-year in this study can partly be explained by the relatively small difference in effect on the reduced number of cigarettes between the intervention and control groups, and the high health care and productivity costs in this population. Benefits or costs due to changes in tax revenue are not included in a cost-effectiveness analysis but could be a topic of interest in social cost-benefit analyses.

Cessation rates in our control group were higher than those in the studies referenced for power analysis. This might be due to differences in target groups between the studies: we studied cancer survivors, whereas the referenced studies either focused on the general population [24] or cancer survivors with a comorbid problem such as drinking or depression [36]. The similar effect on SC of the MyCourse and control groups might be due to several factors. Notably, twice as many participants in the control group reported the use of nicotine replacement therapy, suggesting that this might have influenced SC rates. The control group participants may have had an increased need for additional support. As this was a pragmatic trial, both groups were provided with the contact details of a free national telephone helpline (in Dutch: Rokeninfo-lijn), which could help find participants additional support if the current intervention was deemed insufficient. Furthermore, to recruit participants, a dedicated website and social media campaign was in place, aimed at informing cancer survivors of the short-term benefits of SC after a cancer diagnosis, emphasizing an accepting tone to reduce possible feelings of guilt, and ultimately guide them to participate in the study. Other contributing factors might have been related to the fact that over the course of the study period, participants received multiple reminder emails and telephone calls from the researchers to fill out the survey at the respective follow-up measurement waves. Although these calls were kept as short as possible, some participants might have experienced those as part of the intervention, feeling supported by them, which could have influenced SC rates. To summarize, the low-threshold provision of psychoeducation, offered in an accepting manner, encouragement to seek support provided in recruitment materials and the information brochure, repeated reminders, and increased use of nicotine replacement therapy may have been sufficient to support many participants in their SC efforts. This should be evaluated in future studies.

To considerably increase SC rates among cancer survivors compared with control groups, intensive and well-implemented programs are needed. It is possible that cancer survivors require digital interventions with more guidance, as suggested by a meta-analysis that found that only nurse-delivered SC interventions moderated effectiveness among cancer survivors [17]. Guidance might also help improve adherence, as the median number of log-ins (median 3) was lower than the recommended (almost) daily use of the intervention over the course of 4 weeks. The period between the first and last log-in came close to use as intended, with a median of 24 days.

### Strengths and Limitations

The findings of this study should be interpreted in light of their strengths and limitations. A strength of this study is that the evaluation was conducted in a real-world setting: recruitment was done through both offline and web-based channels, which will also be used for the intervention’s future implementation. The difference in the number of people who completed the screening questionnaire and those who were eligible might seem to show a large selection, but this could be due to our web-based recruitment strategies, which attracted many interested people with no history of cancer on the website as well. This study recruited cancer survivors from a range of cancer types. Several sensitivity analyses were used to corroborate these findings. Missing data were dealt with using multiple imputations. We attempted to control for possible social desirability of reported smoking behavior by including MCSDS scores in our models. Limitations include the fact that participants were not blinded to their intervention allocation. Most of the participants were women (82.6%); therefore, these results might not be generalizable to men. Self-reported smoking status was not biochemically verified in this study, although self-reports in the general population generally showed good validity, 2 studies among cancer survivors found falsification rates of 48% [48] and 80% [49], whereas a third (substantially smaller study) among thoracic cancer survivors showed relatively good agreement between self-reported and biochemically validated smoking status [50]. Adherence was a limitation in this study and might have influenced the effects in the MyCourse group. The effects were limited in size, and our final sample size was somewhat lower than anticipated. As for any RCT, it remains possible that a true effect was not found in this study (type 2 error). Both the main outcome measure (SC during a single week) and the length of follow-up time (a single year) may have masked the possible long-term impact of sustained cessation on long-term health care use and productivity losses. Now it remains for future studies to evaluate whether sustained SC over several years would have contributed to a reduction in health care and societal costs in the long run.

### Conclusions

There was no evidence that at 6 months, the digital MyCourse intervention had a differential effect on cessation rates among cancer survivors compared with a web-based noninteractive information brochure; both conditions led to approximately a quarter of the cancer survivors quitting smoking. The number of cigarettes smoked was reduced by 50% in both groups. At 12 months, assignment to the MyCourse group was associated with a greater reduction in the number of smoked cigarettes at higher costs and higher satisfaction scores compared with the control group. It should be further investigated how to achieve considerably higher quit rates, but this study provides an indication that it is possible to achieve somewhat higher cigarette reduction rates with the help of a digital SC intervention. Although both interventions were low-cost, the noninteractive information brochure was more likely to be economically sustainable.

## Appendix

Multimedia Appendix 1 Additional information on the Methods section.

Multimedia Appendix 2 Attrition and satisfaction with the intervention.

Multimedia Appendix 3 Cost-effectiveness planes and cost-effectiveness acceptability curves after Winsorization.

Multimedia Appendix 4 CONSORT-eHEALTH checklist (V 1.6.1).

## Abbreviations

AUDIT: Alcohol Use Disorders Identification Test
CEAC: cost-effectiveness acceptability curve
CONSORT: Consolidated Standards of Reporting Trials
EQ-5D-5L: 5-level EuroQoL 5
FTND: Fagerström Test for Nicotine Dependence
GLMM: Generalized Linear Mixed Model
ICER: incremental cost-effectiveness ratio
IRR: incidence rate ratio
MCSDS: Marlowe-Crowne Social Desirability Scale
OR: odds ratio
QALY: quality-adjusted life year
RCT: randomized controlled trial
RR: relative risk
SC: smoking cessation
TLFB: timeline Followback
ZUF-8: Fragebogen zur Messung der Patientenzufriedenheit (Patient Satisfaction Questionnaire)
