# Supplementary material for: Effectiveness, Cost-effectiveness, and Cost-Utility of a Digital Smoking Cessation Intervention for Cancer Survivors: Health Economic Evaluation and Outcomes of a Pragmatic Randomized Controlled Trial
Source: J Med Internet Res. 2022 Mar 17;24(3):e27588. doi: 10.2196/27588 (PMC9491833; doi:10.2196/27588)
Supplement: Multimedia Appendix 1 [file jmir_v24i3e27588_app1.docx]

Supplementary material for

“Cost-effectiveness of a digital smoking cessation intervention for cancer survivors: health economic evaluation alongside a pragmatic randomized controlled trial”

Additional information on Methods

*Intervention*

The peer support platform was a moderated bulletin board where participants could provide tips and share experiences on SC with other participants. Participants could choose to use the intervention whenever they wanted for the duration of the study, but were encouraged to log in daily for at least four weeks, e.g. using automated intervention reminder emails. They were also encouraged to engage their own social network through semi-automated email options throughout the program, for example sharing their quit plan or sharing their answers to an exercise about ways in which the participant would like to be supported by their social network in their SC efforts. Questions about the intervention could be directed at the research staff through email or telephone, but for information on more intensive/guided SC support participants were referred to the national SC information line (www.Rokeninfo.nl).

*Sensitivity Analyses*

Missing data for primary and secondary outcome measures were imputed using a second package: the Amelia 2-package. Inspection of the distributions of the imputed data showed that the mice-package predictive mean matching method outperformed the Amelia 2-package. This is not surprising as the Amelia 2-package can only handle multivariate normally distributed data, while tobacco use variables can be considered a form of count data and usually do not approach a multivariate normal distribution. We repeated the main analyses on both the Amelia 2-imputed data and the respondent only data (i.e., data without imputation). For evaluation of number of cigarettes we also conducted a Poisson regression with correction for overdispersion, this is another recommended approach for substance use data (Atkins et al. 2013). For the incremental costs analyses we winsorized the most extreme healthcare costs at the 95^th^ percentile in a sensitivity analysis and based QALYs on SF6D scores instead of the EQ-5D-5L. For the cost-effectiveness analyses, we performed an alternative calculation of the reduction in pack-years by taking into account the reduction at 3, 6 and 12 months and not only at 12 months.
