# Supplementary material for: Effectiveness, Cost-effectiveness, and Cost-Utility of a Digital Smoking Cessation Intervention for Cancer Survivors: Health Economic Evaluation and Outcomes of a Pragmatic Randomized Controlled Trial
Source: J Med Internet Res. 2022 Mar 17;24(3):e27588. doi: 10.2196/27588 (PMC9491833; doi:10.2196/27588)
Supplement: Multimedia Appendix 2 [file jmir_v24i3e27588_app2.docx]

Supplementary material for

“Cost-effectiveness of a digital smoking cessation intervention for cancer survivors: health economic evaluation alongside a pragmatic randomized controlled trial”

Supplementary Table 5. Attrition and satisfaction with the intervention

| **Measures** | | **Primary and secondary meausures** | **Total (N=165)** | **Intervention (N=83)** | **Control (N=82)** |
| --- | --- | --- | --- | --- | --- |
| **3 month follow-up** | | | | | |
|  | missing, n (%) |  |  |  |  |
|  | | cessation | 48 (29.1) | 28 (33.7) | 20 (24.3) |
|  | | number of cigarettes | 48 (29.1) | 28 (33.7) | 20 (24.3) |
|  | | Fagerstrom | 49 (29.7) | 28 (33.7) | 21 (25.6) |
|  | follow-up period, mean days (SD) |  | 101.6 (15.1) | 100.3 (11.7) | 102.8 (17.7) |
|  | ZUF score |  | - | 21.4 (4.61) | 17.3 (6.11) |
| **6 month follow-up** | | | | | |
|  | missing, n (%) | | | | |
|  | | cessation | 52 (31.5) | 24 (28.9) | 28 (34.1) |
|  | | number of cigarettes | 52 (31.5) | 24 (28.9) | 28 (34.1) |
|  | | Fagerstrom | 54 (32.7) | 26 (31.3) | 28 (34.1) |
|  | follow-up period in days, mean (SD) |  | 195.6 (21.9) | 197.0 (21.8) | 194.0 (22.0) |
| **12 month follow-up** | | | | | |
|  | missing, n (%) | | | | |
|  | | cessation | 53 (32.1) | 27 (32.5) | 26 (31.7) |
|  | | number of cigarettes | 53 (32.1) | 27 (32.5) | 26 (31.7) |
|  | | Fagerstrom | 56 (33.9) | 29 (34.9) | 27 (32.9) |
|  | follow-up period in days, mean (SD) |  | 374.1 (24.3) | 375.9 (17.3) | 372.3 (29.8) |
