# Supplementary material for: Effectiveness, Cost-effectiveness, and Cost-Utility of a Digital Smoking Cessation Intervention for Cancer Survivors: Health Economic Evaluation and Outcomes of a Pragmatic Randomized Controlled Trial
Source: J Med Internet Res. 2022 Mar 17;24(3):e27588. doi: 10.2196/27588 (PMC9491833; doi:10.2196/27588)

Supplementary material for

“Cost-effectiveness of a digital smoking cessation intervention for cancer survivors: health economic evaluation alongside a pragmatic randomized controlled trial”

Figures: Cost-effectiveness planes and cost-effectiveness acceptability curves after winsorization and after taking into account a gradual decline in pack-years

Figure S1. Cost-effectiveness planes and cost-effectiveness acceptability curves after winsorization of costs


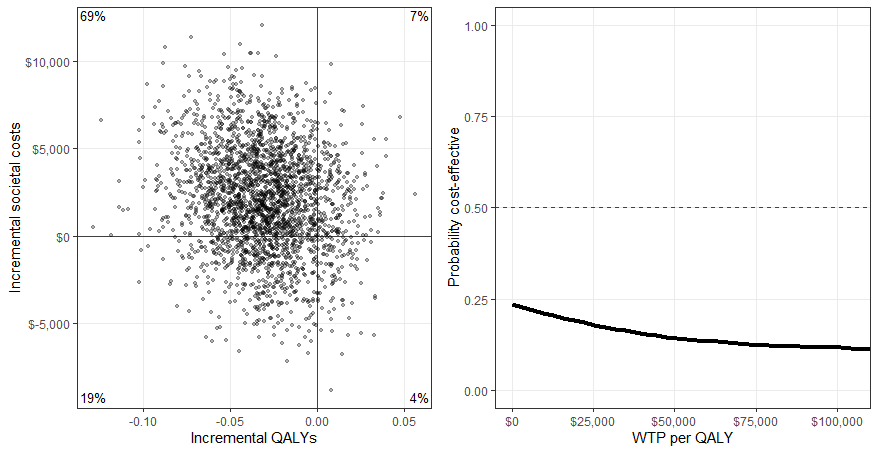


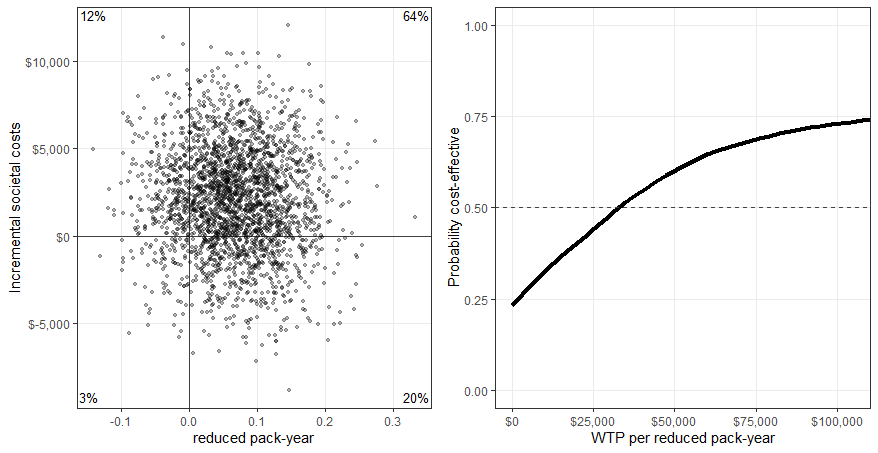


Figure S2. Cost-effectiveness planes and cost-effectiveness acceptability curves after taking into account a gradual decline in pack-years


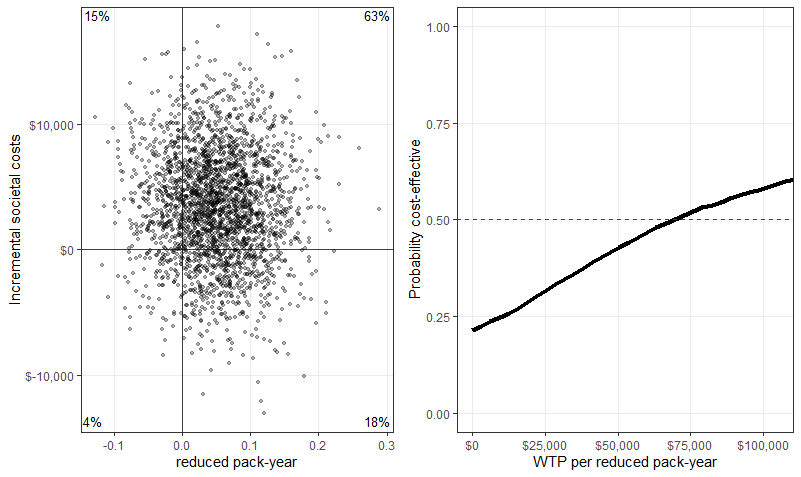

Supplement: Multimedia Appendix 3 [file jmir_v24i3e27588_app3.docx]
